# Whole-genome sequencing of two endophytic bacteria from immature cones of Northern white cedar (*Thuja occidentalis*)

**DOI:** 10.1128/mra.01209-25

**Published:** 2026-03-12

**Authors:** Girish Kumar, Han Ming Gan, Peter Wengert, Emily Kearney, André O. Hudson, Michael A. Savka

**Affiliations:** 1 Program in Biotechnology and Molecular Bioscience, The Thomas H. Gosnell School of Life Sciences, Rochester Institute of Technology 6925, Rochester, New York, USA; 2 Patriot Biotech Sdn Bhd, Subang Jaya, Malaysia; University of Strathclyde, Glasgow, United Kingdom

**Keywords:** bacterial endophyte genomes, white cedar cone bacteria

## Abstract

We report the whole-genome sequences of two bacterial endophytes isolated from immature cones of white cedar (*Thuja occidentalis*). Genome-based taxonomic analysis identified the isolates as *Pantoea trifolii* and *Pseudomonas* sp. These genome sequences provide a resource for studies on plant-microbe interactions and the roles of endophytic bacteria in conifer reproductive tissues.

## ANNOUNCEMENT

Northern white cedar (*Thuja occidentalis*) is a coniferous evergreen tree. Its lightweight and decay-resistant wood is important and commonly used to manufacture products that are exposed to water and soil (1). In this study, we successfully isolated and sequenced two bacterial endophytes associated with immature cones of white cedar.

White cedar samples were collected from the Schoodic area of Maine (44.345955,-68.056299) and subjected to surface sterilization using a 20% Clorox solution (sodium hypochlorite), followed by two washes with sterile distilled water. Immature cones were dissected and placed into flasks using sterile techniques and incubated in tryptic soy broth (TSB) at 28°C with continuous shaking at 200 rpm for 3 days. The resulting culture was plated onto tryptic soy agar and incubated under the same conditions, except for no shaking, to facilitate endophyte isolation. Two distinct colonies with unique morphologies were subsequently identified through two sequential single colony purifications by plate streaking after 72 h of incubation each. To prepare strains for gDNA extraction, a single colony was sub-cultured in 50 mL of liquid TSB for 48 h at 28°C with constant shaking at 150 rpm.

Bacterial DNA was extracted from 25 mL of cell culture using the E.Z.N.A. Bacterial DNA Kit (Omega Bio-Tek, Norcross, GA, USA), followed by library preparation using the Nextera XT Library Preparation Kit (Illumina, San Diego, CA, USA) and sequencing on the Illumina MiSeq (2 × 300 bp run configuration) (Illumina, San Diego, CA, USA) instrument’s default adapter-trimming settings enabled. Raw reads underwent quality control processing using fastp version 0.23.2 (2) and were assembled *de novo* into contigs using SPAdes version 3.15.4 (3). Genome annotation was performed automatically upon upload using the NCBI Prokaryotic Genome Annotation Pipeline version 6.10 (http://www.ncbi.nlm.nih.gov/genome/annotation_prok/) (4). FastANI version 1.33 (5) was used to calculate the pairwise average nucleotide identity (ANI) of the assembled genome against the representative genomes. For phylogenomic construction, the genome of each strain was analyzed using GToTree version 1.6.41 (5), which predicts the proteome and identifies a set of conserved single-copy bacterial marker genes. These markers were aligned, concatenated, and used to construct an approximately maximum likelihood tree with FastTree version 2.1.11 (6). All software was run with default parameters unless otherwise specified.

**Fig 1:**
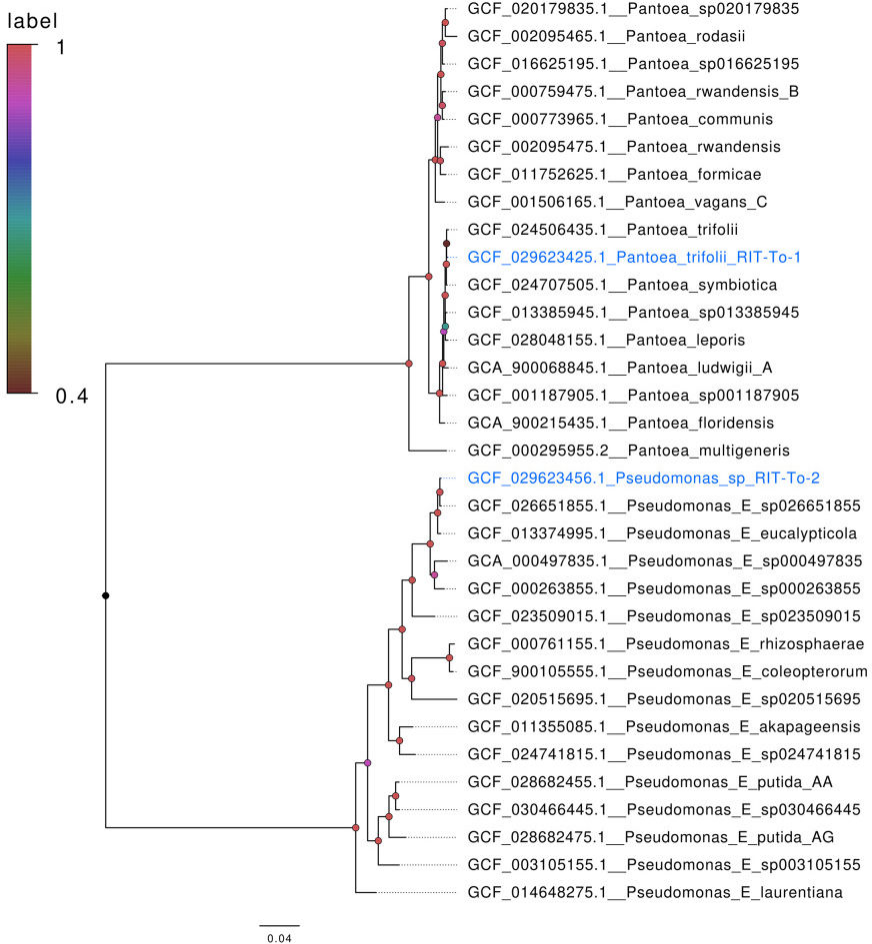
Approximate maximum likelihood tree showing the evolutionary relationships between the isolates sequenced in this study and their closest related species. Strains from this study are highlighted in blue. Branch lengths represent the number of substitutions per site, and node colors indicate Shimodaira–Hasegawa-like approximate likelihood ratio test support values.

The endophyte species (*Pantoea trifolii* RIT-To-1 and *Pseudomonas* sp. RIT-To-2) clustered within their respective genera in the approximate maximum likelihood tree (Fig. 1) (6, 7). In addition, their genome assembly and annotation statistics fall within the expected ranges for their respective genera (Table 1). *Pseudomonas* sp. is recognized as a plant growth-promoting bacterium and a biological control agent (8, 9). In 2023, Strain RIT-To-1 was initially assigned as *Candidatus Pantoea symbiotica* by NCBI based on the best sequence match hit. However, this designation has been revised following the recent formal description of genome sequencing of *Pantoea trifolii* MMK2^T^ (10). Strain RIT-To-1 exhibited 95.51% ANI to *P. trifolii* strain MMK2^T^, supporting its reassignment to this formal species. Consistently, pairwise ANI analysis between *Candidatus Pantoea symbiotica* and *P. trifolii* strain MMK2^T^ showed 95.74%, indicating that they represent the same genomic species.

**TABLE 1:** Genome annotation information for the two white cedar-associated bacteria

Sample	Taxonomic designation	No. of raw reads	No. of bases	SRA accession	Accession number	Best type strain hit (accession number)	ANI (%)	Assembly size (bp)	Coverage (×)	No. of contigs	*N*_50_ (kb)	GC (%)	No. of genes
RIT-To-1	*Pantoea trifolii*	2,947,086	692,989,144	SRR22752009	GCA_029623425.1	*Pantoea trifolii* (GCF_024506435.1)	95.51	5,138,831	121	40	570	54.5	4,780
RIT-To-2	*Pseudomonas* sp.	2,777,022	658,948,306	SRR22752008	GCA_029623465.1	*Pseudomonas eucalyticola* (GCA_013374995.1)	92.27	6,701,612	97	114	153	63.06	6,177

## Data Availability

The whole-genome sequences reported in this article were deposited in GenBank, and accession numbers for each SRA and whole-genome assembly are available in Table 1.

## AFTER EPUB

*[This article was published on 12 March 2026 with an error in the author byline. The author byline was corrected in the current version, posted on 16 March 2026.]*
